# Supplementary material for: Frame-wise multi-echo distortion correction for superior functional MRI
Source: Imaging Neurosci (Camb). 2026 May 29;4:IMAG.a.1262. doi: 10.1162/IMAG.a.1262 (PMC13224312; doi:10.1162/IMAG.a.1262)
Supplement: Supplementary Material [file IMAG.a.1262_supp.pdf]

Supplementary Materials

1 Rigid-body alignment parameters for head motion data

Supplementary Table 1 Average (Std. Dev.) of alignment parameters for each head position

Task	rx (deg)	ry (deg)	rz (deg)	tx (mm)	ty (mm)	tz (mm)
Neutral	-0.09 (0.04)	-0.15 (0.08)	-0.08 (0.09)	-0.17 (0.13)	-0.06 (0.04)	0.02 (0.07)
Rotate +z	-1.54 (0.10)	-1.47 (0.05)	**14.96 (0.08)**	-2.26 (0.23)	-0.69 (0.04)	-2.94 (0.31)
Rotate -z	0.99 (0.07)	-1.90 (0.08)	**-9.78 (0.05)**	-3.84 (0.05)	-0.26 (0.04)	-1.52 (0.09)
Rotate +x	**10.64 (0.27)**	-2.510 (0.15)	0.85 (0.07)	-0.98 (0.10)	4.93 (0.18)	3.78 (0.17)
Rotate -x	**-13.73 (0.24)**	-2.799 (0.07)	-0.94 (0.16)	-2.45 (0.25)	-4.22 (0.07)	0.71 (0.14)
Rotate +y	-1.38 (0.05)	**-10.79 (0.06)**	21.44 (0.06)	2.72 (0.08)	-2.15 (0.04)	-5.35 (0.15)
Rotate -y	0.09 (0.09)	**8.58 (0.22)**	-18.85 (0.12)	-5.79 (0.13)	-1.21 (0.04)	-3.31 (0.07)

2 Anatomical alignment metrics comparing MEDIC and TOPUP distortion correction methods

Supplementary Table 2 Alignment metrics MEDIC vs. TOPUP

Metric	MEDIC	TOPUP	t-statistic	p-value	df
T1w R^2 Spotlight	0.068 (0.007)	0.066 (0.008)	7.133	<0.001	184
T2w R^2 Spotlight	0.083 (0.010)	0.081 (0.011)	6.124	<0.001	184
T1w R^2	0.063 (0.028)	0.060 (0.028)	11.284	<0.001	184
T2w R^2	0.457 (0.053)	0.454 (0.056)	2.729	0.007	184
T1w Grad. Correlation	0.43 (0.028)	0.414 (0.036)	11.727	<0.001	184
T2w Grad. Correlation	0.637 (0.04)	0.638 (0.054)	-0.371	0.711	184
T1w NMI	0.872 (0.029)	0.872 (0.029)	-0.106	0.915	184
T2w NMI	0.836 (0.026)	0.838 (0.026)	-1.985	0.049	184
Gray/White Matter AUC	0.692 (0.031)	0.686 (0.036)	6.598	<0.001	184
Brain/Exterior AUC	0.735 (0.035)	0.729 (0.034)	11.488	<0.001	184
Ventricles/White Matter AUC	0.829 (0.057)	0.829 (0.062)	-0.058	0.954	184
Cerebellum/ Exterior AUC	0.607 (0.041)	0.596 (0.049)	5.073	<0.001	184

3 TOPUP field map for high motion data

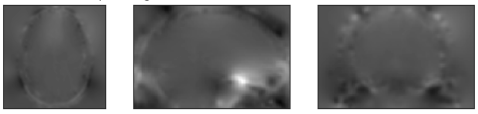

Supplemental Figure 1 TOPUP field map for high motion data. Spin-echo field maps (TR: 8 s, TE: 66 ms, 72 Slices, FOV: 110x110, Voxel Size: 2.0mm) were collected prior to high motion data collection to simulate a typical acquisition of a field map. Field map data was acquired when the head was in the neutral position. Scans were subsequently passed into TOPUP for B0 field estimation using TOPUP's default settings. The same field map was applied to all frames for correction, regardless of head position, after motion correction to a reference frame.

4 Additional exemplar RSFC seed maps

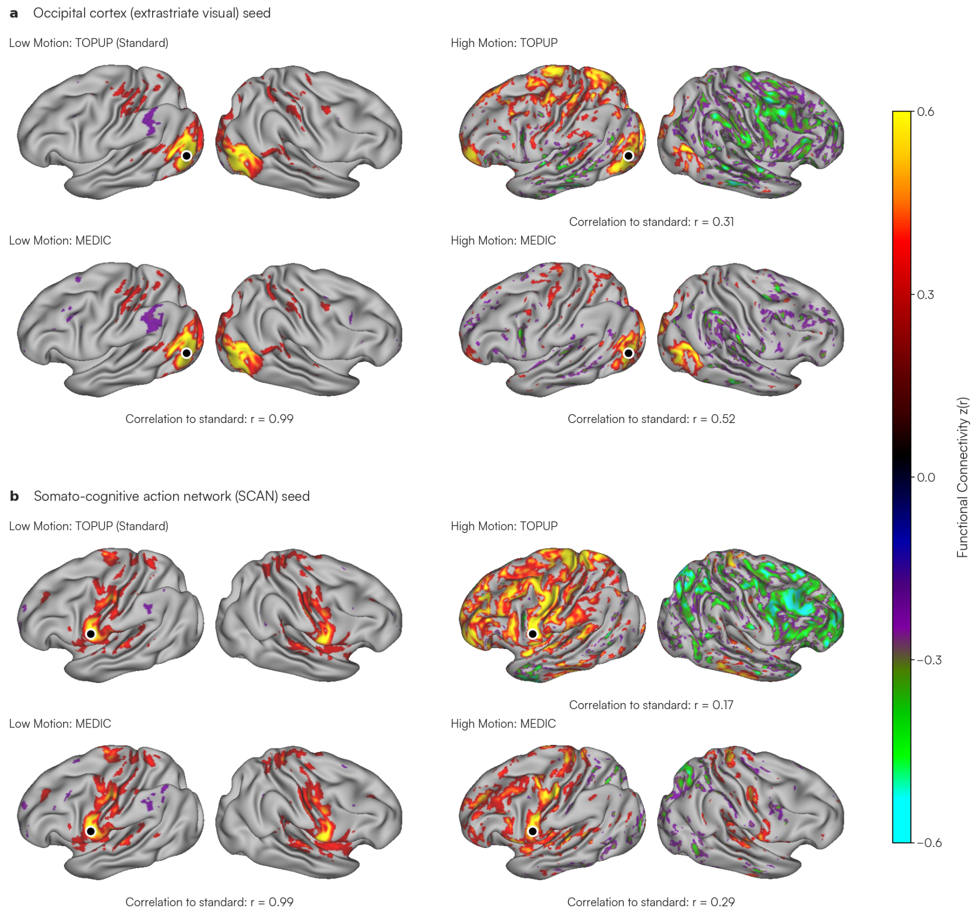

Supplemental Figure 2 Comparison of distortion correction methods (MEDIC vs. TOPUP) in additional exemplar RSFC seed maps. To demonstrate the consistency of MEDIC's performance across diverse functional networks, RSFC seed maps were generated for the same participant using two additional exemplar seeds in (a) occipital cortex (extrastriate visual) and (b) somato-cognitive action network (SCAN). All maps are Fisher z-transformed and thresholded at $|z(r)| > 0.25$ for visualization. Correlations between the low-motion TOPUP standard and each method are displayed below the respective seed maps.

5 MEDIC field maps can measure respiration induced B0 field changes

One well known phenomenon is the effect of respiration on the B0 field (Pfeuffer, Van de Moortele, Ugurbil, Hu, & Glover 2002). As the participant inhales and exhales, the shifting of organs within the thoracic and abdominal regions, coupled with alterations in the oxygenation levels of the breathed-in gas, leads to global oscillations in the B0 field. These global oscillations, through dynamic field mapping, can be measured by MEDIC field maps. We aimed to examine whether respiration could be measured solely with a MEDIC dynamic field map, through averaging of all voxels in the field map and high pass filtering the resultant signal (4th order butterworth, 0.15 Hz cutoff frequency) to obtain an estimation of the participant's respiration signal.

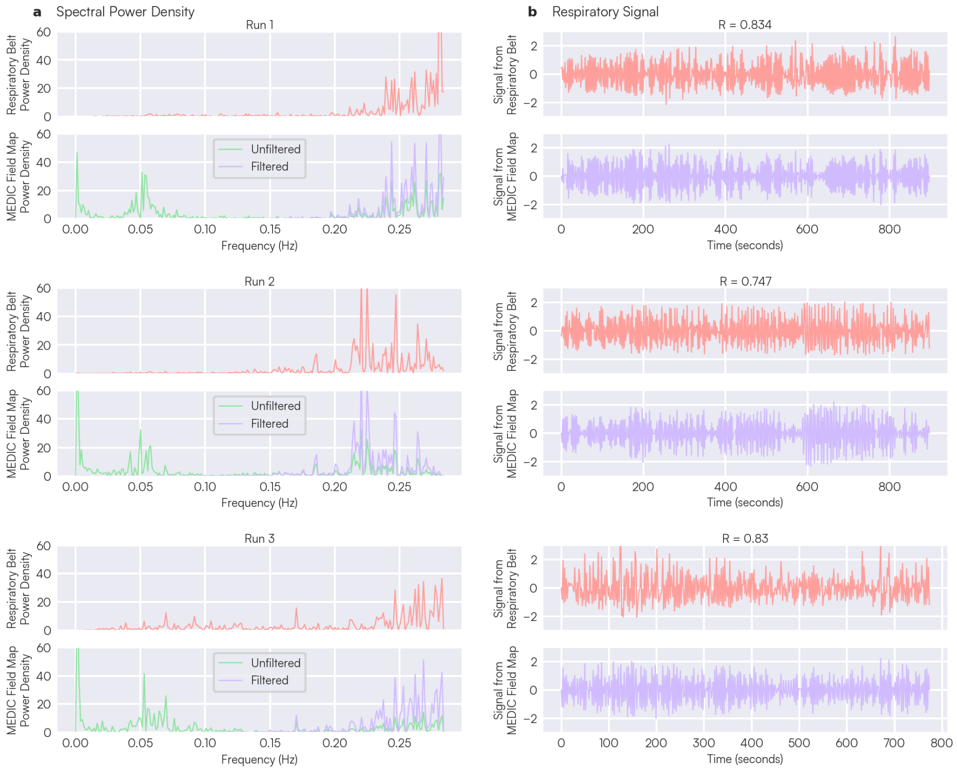

Supplemental Figure 3 Comparison of respiration signal from respiratory belt against respiration signal extracted from MEDIC field maps across 3 runs of the same participant. All data was mean/std. dev. normalized before each analysis. (a) Power spectral density of signal from respiratory belt and MEDIC field maps. Red spectral plot indicates spectral frequency content collected from respiratory belt data from each run. Green and purple spectral plots indicate the frequency content from the average field map time series before and after filtering with a high pass filter for each run (butterworth filter; 4th order; cutoff frequency 0.15 Hz). (b) Signal from the respiratory belt (red) and filtered signal (purple) from the MEDIC field across each run. R values above each plot run indicates the correlation between the two signals.

MEDIC field maps were computed for a single participant with three runs of ME-EPI data with corresponding respiration belt data for comparison Supplemental Fig. 3. MEDIC field maps contain spectral frequency content in the 0.2 Hz to 0.3 Hz band, which generally corresponds to frequencies associated with respiration (˜12 - 20 breaths per minute). Filtering the MEDIC field map signal with a high pass filter (4th order butterworth, 0.15 Hz cutoff frequency) isolates these frequencies for comparison to the respiration signal acquired from the respiratory belt. This filtered signal has a high correlation to the respiratory belt signal across each run (Run 1: R = 0.834; Run 2: R = 0.747; Run 3: R = 0.830) indicating successful extraction of the respiration signal from a MEDIC field map.

This capability offers a synchronized physiological monitoring feature that is inherently time-locked to imaging data. As a result, MEDIC can provide either a redundant or supplemental means of collecting respiration signals during scanning sessions. This is especially crucial given the complexities and challenges of capturing respiration data due to issues like respiratory belt clipping and/or malfunctions. Moreover, the respiration signal used in MEDIC field maps may be used to improve current data pre-processing and analysis methods, thereby enhancing data quality.

References

Pfeuffer, J., Van de Moortele, P.-F., Ugurbil, K., Hu, X., Glover, G.H. (2002). Correction of physiologically induced global off-resonance effects in dynamic echo-planar and spiral functional imaging. *Magnetic Resonance in Medicine*, *47*(2), 344–353, https://doi.org/10.1002/mrm.10065
